# Innate immune sensing of a growing tumor in vivo occurs via the host sting pathway and involves mitochondrial DNA transfer from cancer cells to DCs

**DOI:** 10.1186/2051-1426-1-S1-O19

**Published:** 2013-11-07

**Authors:** Seng-Ryong Woo, Michael Leung, Michael Furdyna, Thomas F Gajewski

**Affiliations:** 1 Pathology, University of Chicago, Chicago, IL, USA; 2 Medicine, University of Chicago, Chicago, IL, USA

## 

Spontaneous T cell responses against tumors occur frequently. However, the mechanisms by which innate immune responses become induced in response to cancer, and how they can bridge to T cell priming against tumor antigens, have been poorly understood. We recently showed that CD11c+ cells produce IFN-β after tumor implantation and this IFN-β plays a critical role in cross-priming of endogenous anti-tumor CD8+ T cells in vivo. Using knockout mice lacking candidate innate immune sensing molecules known to be able to induce type I IFN production in APCs, we have identified a critical role for the host STING pathway in tumor recognition, spontaneous T cell priming, and tumor control in vivo. As STING has been implicated in cytosolic DNA sensing, these results implied that tumor-derived DNA might be the critical ligand for effective innate immune activation. Therefore, we questioned whether tumor-derived DNA might be transferred to host APCs in vivo and lead to STING pathway activation and IFN-β production. To test this notion, three complementary methods were employed. Tumor cells were stained either with the DNA intercalating dye DRAQ5 or the nucleotide analogue Edu and injected into mice. After one day, tumor-infiltrating CD45+CD11c+ cells were analyzed by ImageStream single cell cytometry. In fact, a major fraction of host DCs appeared to acquire tumor-derived DNA using these methods. As a third approach, we utilized a human melanoma xenograft model that allowed interrogation of host DCs for the presence of human DNA sequences using species-specific PCR. This strategy also allowed assessment of both mitochondrial DNA and genomic DNA. Tumors were implanted and DCs were highly purified by flow cytometric sorting. Interestingly, human mitochondrial DNA was clearly detected in sorted mouse CD45+CD11c+ cells. However, human genomic DNA sequences were not detected. To further investigate whether mitochondria transfer actually occurred, we specifically labeled mitochondria in B16 melanoma using viral transduction to express a mitochondria-specific protein conjugated with GFP and transplanted these tumors into mice. In fact, GFP transfer was detected in the host APCs in a morphology that resembled whole mitochondria. The host CD11c+ cells also showed phospho-IRF3 induction and IFN-β production ex vivo. Our data suggest that tumor-derived mitochondrial DNA can be transferred to host APCs in vivo and may serve as the major ligand for STING pathway activation, IFN-β production, and subsequent T cell activation against tumors.

